# Endophytic bacteria associated with wild-type banana seed (*Musa balbisiana*): whole genome sequencing

**DOI:** 10.1128/MRA.00650-23

**Published:** 2023-11-03

**Authors:** Girish Kumar, Han Ming Gan, Hailey Popielarz, Julia Steele, Anutthaman Parthasarathy, André O. Hudson, Michael A. Savka

**Affiliations:** 1 Thomas H. Gosnell School of Life Sciences, Rochester Institute of Technology, Rochester, New York, USA; 2 Department of Biological Sciences, Sunway University, Petaling Jaya, Malaysia; 3 Patriot Biotech Sdn Bhd, Subang Jaya, Malaysia; University of Strathclyde, Glasgow, United Kingdom

**Keywords:** *Musa balbisiana*, wild-type banana seed (WTBS), seed endophytes, *Fusarium* wilt

## Abstract

We present the whole-genome sequences of five endophytic bacteria isolated from *Musa balbisiana* seeds. These strains represent five different genera: *Bacillus, Brachybacterium, Enterobacter, Enterococcus,* and *Pantoea*. Among these, three genera (*Bacillus*, *Pantoea*, and *Enterobacter*) were previously recognized for their antagonistic effects against Fusarium wilt, a highly destructive disease that affects banana plants.

## ANNOUNCEMENT

Bananas are one of the most extensively cultivated crops worldwide and are a crucial food source in various developing nations (1). The wild banana (*Musa balbisiana*, family: Musaceae) is native to Southeast Asia, including India, and exhibits characteristics that enable banana to withstand biotic and abiotic stresses (2, 3). Bacteria have been described as being associated with the banana roots (4) and the pseudostem (5); however, endophytes of wild-type banana seed (WTBS) have not been investigated.

In this study, we present the genome sequences of five bacterial endophytes that were obtained from WTBS. The seeds were collected from a single plant at a single location in Chennai, India, which has coordinates of 12.953074°N and 80.149624°E. To obtain culturable bacterial endophytes, seeds were surface sterilized using a 20% solution of sodium hypochlorite (NaOCl) followed by two sequential washes with sterile distilled water. The internal tissue was prepared by dissecting tissues using a scalpel and forceps under sterile conditions. The tissue was then inoculated into tryptic soy broth (TS) medium and incubated at 28°C for 3 days. The resulting culture was serially diluted (10-fold) and plated on TS agar media and incubated under the same conditions to isolate colonies. Five distinct colonies with different morphologies were obtained and were further subcultured twice on the TS agar medium at 28°C for 48 hours to ensure purity.

To extract genomic DNA (gDNA) from each of the five isolates, we used 25 mg of bacterial culture grown in TS broth for 24 hours at 28°C with shaking, employing the E.Z.N.A. bacterial DNA Kit (Omega Bio-Tek, Norcross, GA). For the library preparation, we used the standard procedure outlined in the Nextera XT library preparation kit (Illumina, San Diego, CA). The libraries were diluted to 16 pM prior to sequencing on an Illumina MiSeq (v3 chemistry, 2 × 300 cycles) at the Genomics Lab, Rochester Institute of Technology.

**Fig 1:**
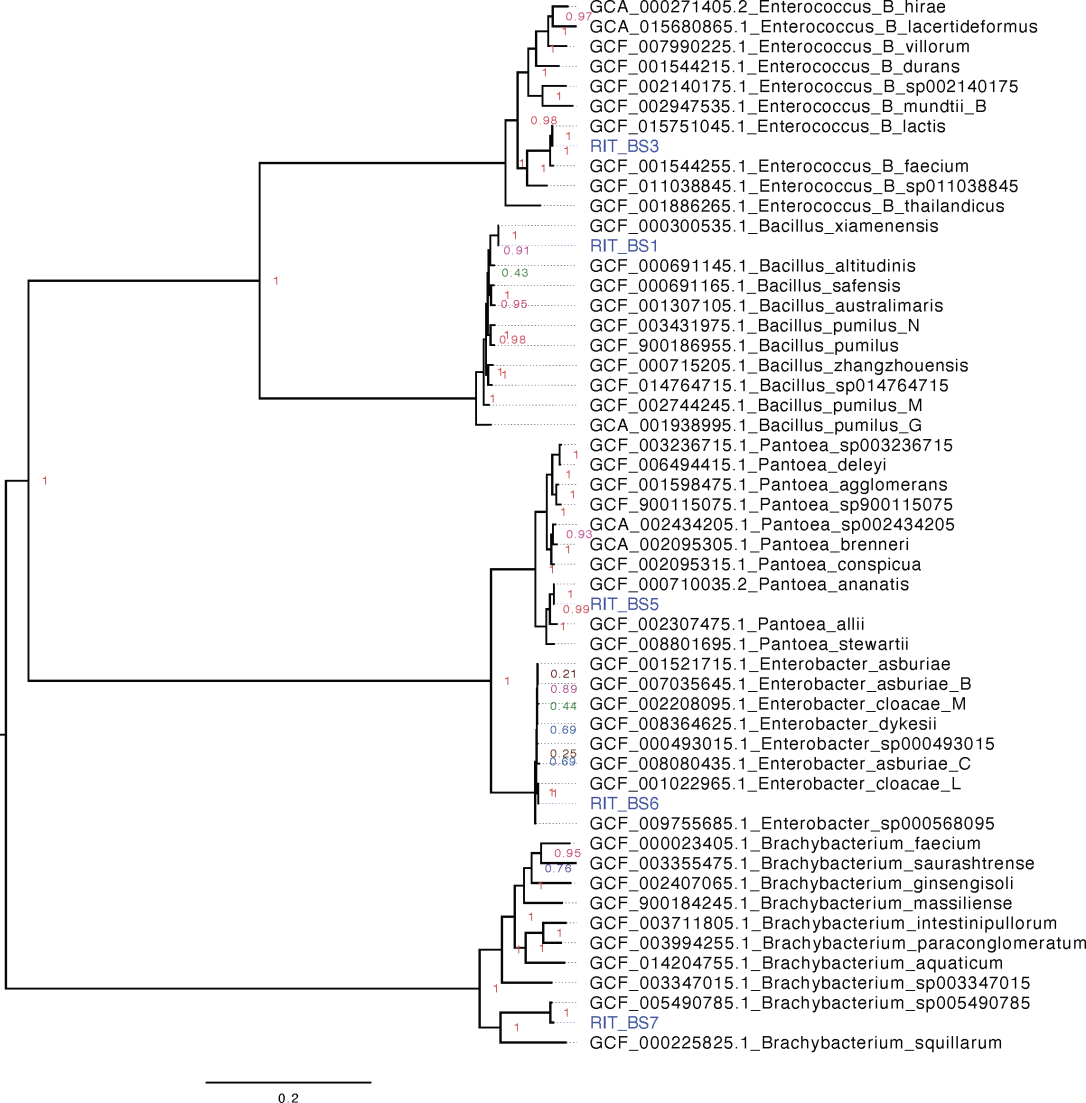
Maximum likelihood tree showing the genome-based evolutionary relationships of banana seed (BS) strains (Blue taxa) with closely related representative strains in the GTDB database. FastANI version 1.33 (10) was used to calculate the pairwise average nucleotide identity of assembled genome against the representative genomes. The phylogenomic tree was constructed with FastTree version 2.1.10 (11) using a concatenated alignment of 74 single-copy bacterial genes produced from the GTOTree (12) using default parameters. Briefly, the GTOTree version 1.6.31 identified and aligned 74 single-copy bacterial genes from each genome assembly using hmmsearch version 3.3.2 (13) and muscle version 3.8.1551 (14), respectively. Branch lengths indicate the number of substitutions per site while node labels indicate Shimodaira-Hasegawa(SH)-like support values.

Default parameters were used for all software unless otherwise specified. The Illumina MiSeq software automatically executed the demultiplexing, FASTQ generation, and adapter trimming of the raw sequence reads. After quality control processing with the program fastp (6) (version 0.23.2), the paired-end raw reads were *de novo* assembled into contigs using the SPAdes genome assembler (7) (version 3.5.0). The annotation of sequences was performed by utilizing the NCBI Prokaryotic Genome Annotation Pipeline (http://www.ncbi.nlm.nih.gov/genome/annotation_prok/ [8]; version 6.5). Among the five genomes that were sequenced, four strains were identified as previously described bacterial species with a pairwise average nucleotide identity of more than 98% (Table 1) while strain RIT_BS7 was classified as an undescribed *Brachybacterium* species (Fig. 1). Strains from the genera *Bacillus*, *Pantoea*, and *Enterobacter* were known to exhibit antagonistic behavior against *Fusarium* wilt (9), which is one of the most harmful diseases affecting banana plants. The data on whole-genome sequencing presented in this study can aid in the identification of genes related to endophyte-mediated resistance in bananas and contribute toward the management of *Fusarium* wilt.

**TABLE 1:** Genome annotation information for bacterial endophytes of wild-type banana seed (*Musa balbisiana*)^*a*^

Sample	Total # of raw reads	SRA accession	Assembly accession	Assigned taxonomy	Reference genome with accession number	% ANI	% cov	Assembly size (bp)	Coverage (X)	# of contigs	N50	Assembly GC content (%)	# of genes	# of tRNA	# of rRNA
RIT_BS1	6,821,562	SRR22031647	GCA_025942125.1	*Bacillus xiamenensis*	*Bacillu xiamenensis* (GCF_000300535.1)	99.61	97.38	3,585,150	452.22	59	188,885	41.42	3,745	64	3
RIT_BS3	6,191,302	SRR22031646	GCA_025942085.1	*Enterococcus faecium*	*Enterococcus lactis* (GCF_015751045.1)	98.37	85.31	2,797,207	547.02	101	84,391	38.11	2,793	51	3
RIT_BS5	6,545,360	SRR22031645	GCA_025942095.1	*Pantoea ananatis*	*Pantoeaananatis* (GCF_000710035.2)	98.70	89.35	4,930,326	291.50	72	260,159	53.03	4,637	67	2
RIT_BS6	4,646,050	SRR22031644	GCA_025942055.1	*Enterobacter asburiae*	*Enterobacter_cloaca* (GCF_001022965.1)	98.42	90.47	5,494,871	248.49	123	372,076	55.26	5,369	75	4
RIT_BS7	4,923,222	SRR22031643	GCA_025942045.1	*Brachybacterium squillarum*	*Brachybacterium sp005490785* (GCF_005490785.1)	97.28	87.11	3,331,992	399.04	117	46,237	73.07	3,057	50	3

^*a*^ %ANI = % average nucleotide identity; % cov= % genome query coverage.

## Data Availability

The whole-genome assemblies and Sequence Read Archive (SRA) accession numbers of the bacterial genomes are presented in Table 1 and can be downloaded from GenBank and SRA, respectively.
